# *Polygonatum sibiricum* Polysaccharides Alleviate LPS-Induced Liver Injury in Chicks by Suppressing Inflammation and Oxidative Stress Through the PPAR Signaling Pathway

**DOI:** 10.3390/antiox14040418

**Published:** 2025-03-31

**Authors:** Yang Li, Jian Li, Xiaowang Liu, Zhili Cheng, Nana Gao, Jungang Kang, Xiaodan Wang

**Affiliations:** 1 College of Traditional Chinese Veterinary Medicine, Hebei Agricultural University, Baoding 071001, China; 20221200095@pgs.hebau.edu.cn (Y.L.); gaonanahbnd2023@163.com (N.G.); kjg1506@163.com (J.K.); 2 Hebei Provincial Animal Husbandry Station, Shijiazhuang 050011, China; 13722868318@163.com (J.L.); 13831190234@163.com (X.L.); sxmzh@163.com (Z.C.)

**Keywords:** lipopolysaccharide, *polygonatum sibiricum* polysaccharides, liver, chicken, gene expression

## Abstract

*Polygonatum sibiricum* polysaccharides (PSPs), plant-derived polysaccharides widely used in the pharmaceutical field, exhibit various biological activities, including anti-inflammatory and antioxidant effects. However, research on their application in chicks remains limited. Therefore, the aim of this study is to investigate the protective mechanism of PSP against liver injury in chicks using an LPS-induced inflammatory model. A total of 200 one-day-old Hy-Line Brown laying chicks were randomly assigned to five groups (40 chicks each): a control group (CON) fed a basal diet, an LPS group, and three PSP groups receiving low (250 mg/L), medium (500 mg/L), and high (1000 mg/L) doses of PSP (PSP250_LPS, PSP500_LPS, and PSP1000_LPS, respectively). The experiment lasted 21 days. During this period, the LPS and PSP groups were intraperitoneally injected with 1500 μg/kg LPS on days 14, 16, 18, and 20, while the CON group received normal saline. On day 21, organs were collected for analysis. The results indicated that PSP treatment significantly reduced the liver and kidney indices that were elevated by LPS (*p* < 0.05) without affecting the indices of the spleen, thymus, or bursa of Fabricius (*p* > 0.05). Histological analysis revealed that PSP alleviated LPS-induced ballooning degeneration and cell swelling in hepatocytes. Furthermore, PSP treatment decreased the levels of ALT and AST and significantly mitigated increases in the pro-inflammatory cytokines IL-1β, IL-6, and TNF-α while enhancing the level of the anti-inflammatory cytokine IL-10 (*p* < 0.05). Transcriptome sequencing of liver samples revealed that LPS significantly altered the expression of 10 genes in the peroxisome proliferator-activated receptor (PPAR) signaling pathway, which were regulated by PSP intervention. qPCR validation supported these findings. Furthermore, biochemical analyses of liver tissue showed that PSP alleviated oxidative stress by affecting levels of SOD, MDA, NADPH, ROS, and H_2_O_2_. In conclusion, PSP may alleviate LPS-induced liver injury in chicks by modulating the PPAR signaling pathway. These findings provide valuable insights for promoting healthy chick rearing and ensuring the safe supply of poultry products.

## 1. Introduction

Intensive poultry farming often faces challenges from frequent pathogenic infections and increasing environmental pollution, which lead to reduced growth performance and impaired immune function in poultry, thereby increasing the risk of disease. Prolonged stress, particularly during the early growth stages, can further inhibit the development of organs such as the liver [1]. Lipopolysaccharide (LPS), a significant pathogenic factor of Gram-negative bacteria, is also known as an ‘endotoxin’ [2]. LPS can bind to the TLR4 receptor, activate the NF-κB pathway, and promote the release of pro-inflammatory mediators [3]. Intraperitoneal or intramuscular injection of LPS in animals can induce inflammation and septic shock, leading to stress reactions such as peripheral vascular collapse [4].

The liver serves as both a target of and an effector organ in response to oxidative stress, significantly impacting the growth potential of animals. LPS-induced oxidative stress can damage the biofilm and function of hepatocytes, leading to hepatomegaly and impaired liver function [5,6,7]. Acute liver failure diminishes the liver’s capacity to eliminate LPS, resulting in an increased production of inflammatory cytokines and subsequent inflammation in the liver [8]. Furthermore, inflammatory mediators triggered by LPS may heighten the risk of neurological complications associated with liver disease [9]. Research has demonstrated that LPS can induce the activation of hepatic stellate cells, causing them to transform into myofibroblasts, thereby playing a significant role in the advancement of liver fibrosis [10]. Therefore, finding effective feed additives to alleviate animal stress, protect the body, and enhance immunity is crucial for improving poultry production efficiency and disease resistance.

Previous research has shown that *Polygonatum sibiricum* polysaccharide possesses a range of biological properties, including anti-inflammatory, antioxidant, and immune-regulatory activities [11,12,13]. A study demonstrated that administering PSP orally can mitigate DNA damage and lipid peroxidation, which are induced by oxidative stress, in a mouse model of cardiac aging triggered by D-lactic acid. Furthermore, PSP can significantly reduce reactive oxygen species (ROS) and malondialdehyde (MDA) levels and increase superoxide dismutase (SOD) levels, suggesting that PSP has antioxidant activity in vivo [14]. PSP can be used as a potential immunosuppressant to protect cyclophosphamide-treated immunosuppressed mice [11]. However, the effect of PSP on LPS-induced liver injury in chicks has not been reported. Therefore, the aim of this study was to investigate the effects of PSP on LPS-induced liver injury in chicks by establishing a model of LPS-induced liver injury and to further analyze the protective mechanisms of PSP through transcriptome sequencing data.

## 2. Materials and Methods

### 2.1. Ethical Statement

The methods utilized in this study involving animals strictly adhere to the guidelines and regulations set forth by the Hebei Agricultural University Committee, ensuring ethical and responsible conduct throughout the research process.

### 2.2. Materials

*Polygonatum sibiricum* polysaccharide (purity 70%), derived from the rhizomes of the *Polygonatum sibiricum* plant, was obtained from Yuanye Bio-Technology Co., Ltd. in Shanghai, China. Female Hy-Line Brown laying hens were supplied by Baoding Xingrui Agriculture and Animal Husbandry Development Co., Ltd., Baoding, China. LPS (purity ≥ 98%, model L2880, serotype O55:B5) was purchased from Sigma, St. Louis, MO, USA.

### 2.3. Grouping of Experimental Animals

A total of 200 healthy 1-day-old female Hy-Line Brown chickens with similar body weights were randomly assigned to five groups, with 40 chickens in each group. All chicks were given unrestricted access to water and food. The experimental groups included the following: control group (CON), LPS group, PSP250_LPS group, PSP500_LPS group, and PSP1000_LPS group. The groups were based on a basal diet with 0, 250, 500, and 1000 mg/L doses of PSP added to the drinking water, respectively. The experiment was conducted over a period of 21 days. On days 14, 16, 18, and 20, the LPS group, PSP250_LPS group, PSP500_LPS group, and PSP1000_LPS group were administered an intraperitoneal injection of 1500 μg/kg LPS. The control group, on the other hand, was administered an equal volume of normal saline.

### 2.4. Diet and Feeding Management of Experimental Animals

The feeding experiment was conducted at the Animal Experimental Farm of Hebei Agricultural University, School of Animal Medicine/College of Traditional Chinese Veterinary Medicine. The experimental diets were formulated in accordance with the nutritional requirements for Chinese chickens (NY/T33-2004) [15]. The diet composition and nutritional levels are presented in Table 1. All groups were provided with the same dietary conditions and were housed in cages under appropriate lighting. During the first week, the temperature was kept at 35 °C and then progressively lowered by 1 °C every two days, ultimately reaching 25 °C. Humidity was kept within the range of 50–70%.

### 2.5. Collection of Liver, Kidney, and Organs of Chicks

The chicks’ livers, kidneys, thymuses, spleens, and bursae of Fabricius were meticulously dissected, with any extraneous blood and fat meticulously trimmed away. After weighing, the organ index for each organ was calculated. Each chick’s liver was then surgically divided in half: one portion was placed in a 10% formalin solution for histological analysis, while the other half was wrapped in tinfoil and immediately frozen in liquid nitrogen. This portion was subsequently stored in an ultra-low-temperature freezer at −80 °C.

Liver index = Liver weight (g) ÷ Body weight (g)
Kidney index = Kidney weight (g) ÷ Body weight (g)
Thymus index = Thymus weight (g) ÷ Body weight (g)
Spleen index = Spleen weight (g) ÷ Body weight (g)
Bursa of Fabricius index = Bursa of Fabricius weight (g) ÷ Body weight (g)

### 2.6. HE Staining of Chick Liver

Chick liver tissue sections collected on day 21 of the experiment were stained and observed using a light microscope (Nikon Eclipse CI, Nikon Inc., Tokyo, Japan).

### 2.7. Detection of Biochemical Indices in Chick Liver

A total of 100 mg of liver tissue was added to a 2 mL centrifuge tube, followed by the addition of 900 μL of normal saline and small steel columns. The tissue was then homogenized using a homogenizer (POLYTRON PT 2500E, Luzern, Switzerland). The homogenate underwent centrifugation at 3500× *g* for a duration of 10 min, after which the supernatant was gathered for the assessment of alanine aminotransferase (**ALT**), aspartate aminotransferase (**AST**), total protein (**TP**), catalase (**CAT**), and glutathione peroxidase (**GSH-Px**) concentrations in the chicks’ liver. The assay kits were obtained from Nanjing Jiancheng Bioengineering Institute Co., Ltd., Nanjing, China, and the detection procedures were performed according to the manufacturer’s guidelines.

### 2.8. Transcriptome Sequencing of Chicken Liver

To gain deeper insights into how PSP impacts the mechanism of LPS-induced liver injury, we performed an analysis of eukaryotic transcriptome sequencing. Our findings suggested that the addition of PSP at a concentration of 500 mg/L had the most significant impact on the liver of the chicks. Consequently, we selected three liver samples from the CON, LPS, and PSP500_LPS groups for high-throughput sequencing. The process involved extracting total RNA from the liver tissue, followed by stringent quality control to ensure accurate assessment of RNA integrity. The qualified RNA fragments were then synthesized into cDNA, and a sequencing library was constructed. All cDNAs were sequenced on an Illumina Novaseq 6000 system provided by LC Bio Technology Co., Ltd., located in Hangzhou, China.

### 2.9. Real-Time Fluorescent Quantitative PCR (qRT-PCR)

Ten differentially expressed genes (**DEGs**) associated with significantly enriched pathways that contribute to the alleviation of LPS-induced liver injury by PSP were selected for analysis using qRT-PCR. A total of 40 mg of liver tissue was homogenized with 500 μL of lysis buffer, followed by the addition of a diluent to mix and extract total RNA. Reverse transcription to synthesize cDNA was conducted according to the manufacturer’s instructions. The relative mRNA expression levels of the target genes were then assessed using qRT-PCR. The primer sequences for the target genes, including *APOC3*, *ACAA1*, *HMGCS2*, *ACSBG2*, *ME1*, *ADIPOQ*, *FABP3*, *FABP4*, *SCD*, and *PCK1*, along with the reference gene *β-actin*, are detailed in Table 2.

### 2.10. Data Analysis and Statistics

After data collation using Excel, SPSS 26.0 software was used for one-way analysis of variance (ANOVA) and Tukey test for multiple comparisons. Data were expressed as ‘mean ± standard deviation (Mean ± SD)’. GraphPad Prism 8.0 was used for data visualization. *p* < 0.05 was considered significant.

## 3. Results

### 3.1. Effect of PSP on LPS-Induced Organ Indices in Chicks

**Figure 1:**
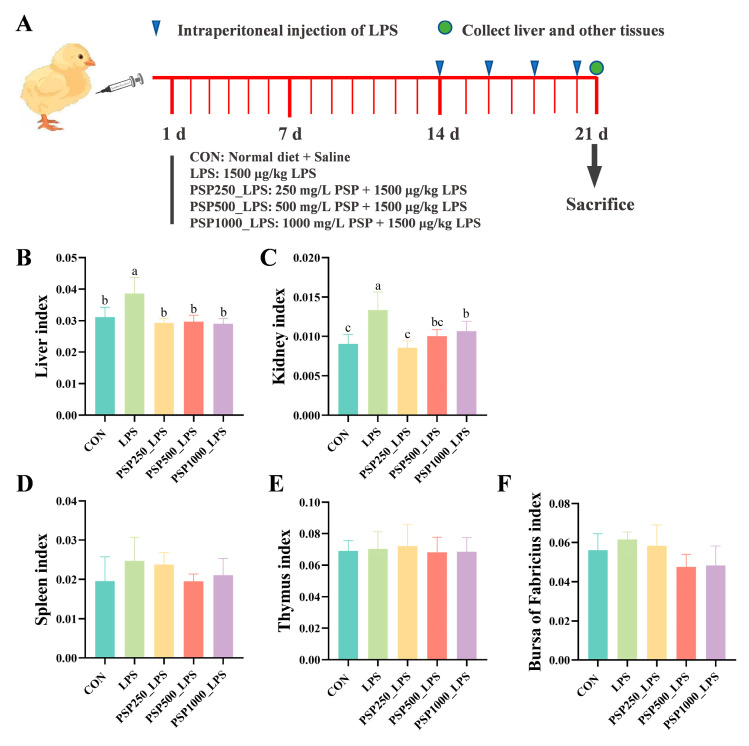
Schematic diagram of the experiment and the effect of PSP on organ indices of LPS-induced chicks. (**A**) Schematic diagram of the experiment. (**B**) Liver index. (**C**) Kidney index. (**D**) Spleen index. (**E**) Thymus index. (**F**) Bursa of Fabricius index. Data columns labelled with different lowercase letters indicate statistically significant differences (*p* < 0.05).

The LPS group exhibited significantly elevated liver and kidney indices in chicks compared to the CON group, indicating potential enlargement of these organs and confirming successful model establishment (*p* < 0.05). The liver and kidney indices were significantly elevated (*p* < 0.05) in chicks after adding different doses of PSP to their drinking water compared to the LPS group (Figure 1B,C). However, PSP treatment did not produce a significant effect (*p* > 0.05) on the indices of the spleen (Figure 1D), thymus (Figure 1E), or bursa (Figure 1F) in the chicks.

### 3.2. Protective Effect of PSP on LPS-Induced Liver Injury in Chicks

**Figure 2:**
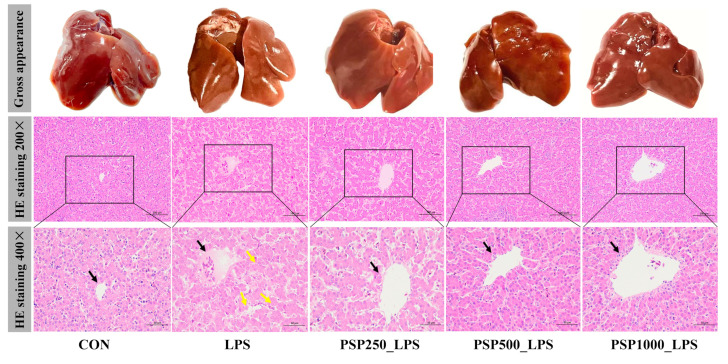
Representative phenotypic photographs and micrographs of liver tissue with H&E staining. Black arrows indicate the central vein of the liver. Yellow arrows indicate hepatocyte necrosis. HE staining showed that PSP could effectively reduce LPS-induced hepatocyte cord structure blur and hepatocyte necrosis.

The liver in the CON group exhibited good light chromaticity, appearing reddish-brown with a smooth surface and normal morphology and size (Figure 2). In contrast, the liver of the LPS group displayed enlargement, a brittle texture, reduced elasticity, and a grayish-yellow color. Compared with LPS group, the liver surface of PSP groups were smoother and displayed more obvious staining. HE staining demonstrated that the LPS group showed a disrupted arrangement of hepatic cords, in contrast to the CON group. The central vein was filled with numerous red blood cells, and the hepatic sinusoids were dilated. Conversely, the liver histopathological changes in the three groups given PSP were significantly improved compared to the LPS group, with well-aligned hepatic cords, reduced inflammatory cell infiltration, and decreased hepatocyte degeneration. Notably, the PSP500_LPS group showed the most pronounced improvement, including the disappearance of erythrocytes in the central vein and a significant reduction in cellular vacuolar degeneration. In conclusion, PSP effectively mitigates LPS-induced liver injury.

### 3.3. Effect of PSP on Liver Biochemical Indices in LPS-Induced Chicks

**Figure 3:**
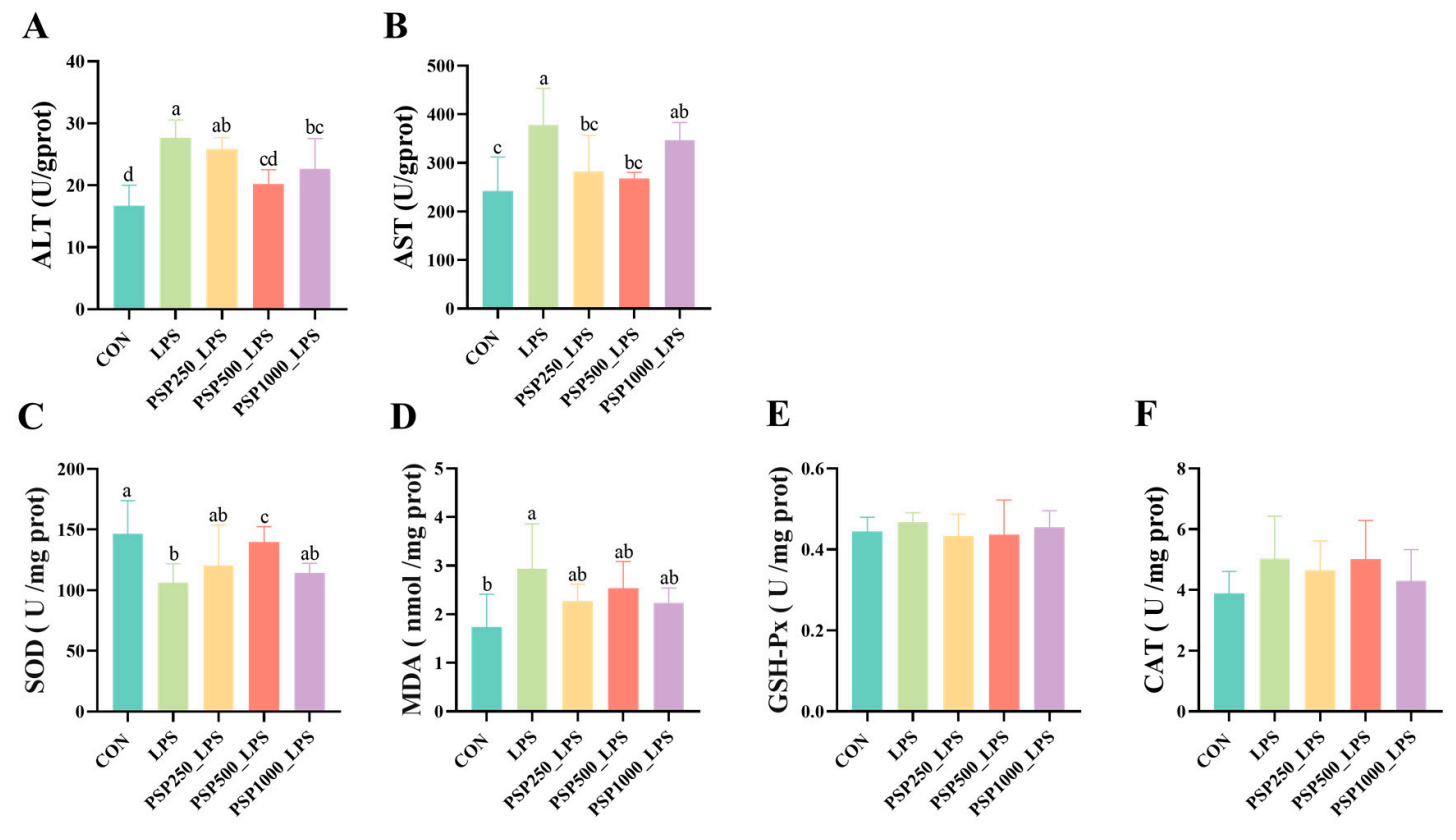
Effect of PSP on biochemical indices of LPS-induced chick liver: (**A**–**F**) are the viability of ALT, AST, SOD, GSH-Px, CAT, and the content of MDA in chick liver, respectively. Data columns labelled with different lowercase letters indicate statistically significant differences (*p* < 0.05).

In comparison to the CON group, the LPS group demonstrated notably elevated levels of ALT and AST (*p* < 0.05). In the PSP dosage groups, there was a tendency for ALT and AST levels to decrease compared to the LPS group (Figure 3A,B). Among them, the PSP500_LPS group showed the most significant relief, with a notable decrease in both ALT and AST levels (*p* < 0.05). This suggests that PSP dosage groups could alleviate LPS-induced liver injury, with the PSP500_LPS group exhibiting the most pronounced effect. Additionally, compared to the CON group, the LPS group had significantly reduced SOD activity (*p* < 0.05) and increased MDA content (*p* < 0.05). The addition of PSP alleviated these trends caused by LPS (Figure 3C,D). However, LPS and PSP had no significant effect (*p* > 0.05) on GSH-Px and CAT activity (Figure 3E,F).

### 3.4. Effect of PSP on LPS-Induced Hepatic Inflammatory Factors in Chicks

**Figure 4:**
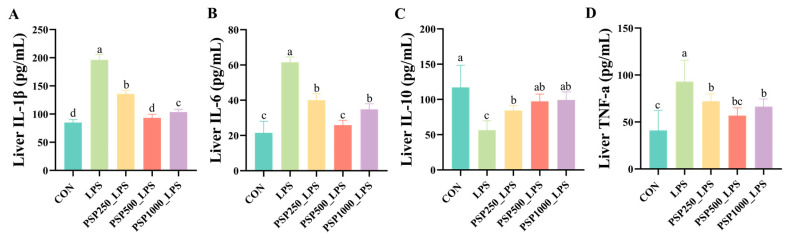
Effect of PSP on LPS-induced hepatic inflammatory factors in chicks: (**A**–**D**) are the hepatic levels of IL-1β, IL-6, IL-10, and TNF-a in chicks, respectively. Data columns labelled with different lowercase letters indicate statistically significant differences (*p* < 0.05).

The levels of IL-1β, IL-6, and TNF-α were significantly elevated in the LPS group compared to the CON group (*p* < 0.05), indicating that LPS induces the release of inflammatory cytokines. In contrast, the administration of varying doses of PSP in the drinking water significantly reduced hepatic inflammatory cytokine levels in chicks compared to the LPS group (*p* < 0.05), demonstrating the anti-inflammatory effects of PSP (Figure 4A,B,D). Furthermore, a notable reduction in IL-10 levels was observed in the LPS group when compared to the CON group (*p* < 0.05). Conversely, treatment with various doses of PSP led to a marked increase in IL-10 levels (*p* < 0.05), thereby reinforcing the anti-inflammatory properties of PSP (Figure 4C).

### 3.5. Transcriptome Data Analysis

Quality analysis of sequencing data. To investigate the mechanisms by which PSP mitigates LPS-induced liver injury in chicks, we selected liver tissues from the CON group, the LPS group, and the PSP500_LPS group for transcriptome sequencing.

As detailed in Table 3, the sequencing data underwent quality analysis, revealing that each sample yielded between 46.1 million and 49.9 million sequences. Following quality control filtering, the valid reads for each sample ranged from 48.7 million to 49.9 million, with valid ratios exceeding 96.37%. The basic mass values of the samples were between 93.01% and 94.33%, and the GC content varied from 46.76% to 49.25%. The stability of the GC percentages, combined with the efficient utilization of sequencing volume, rendered the results satisfactory for subsequent experiments.

**Figure 5:**
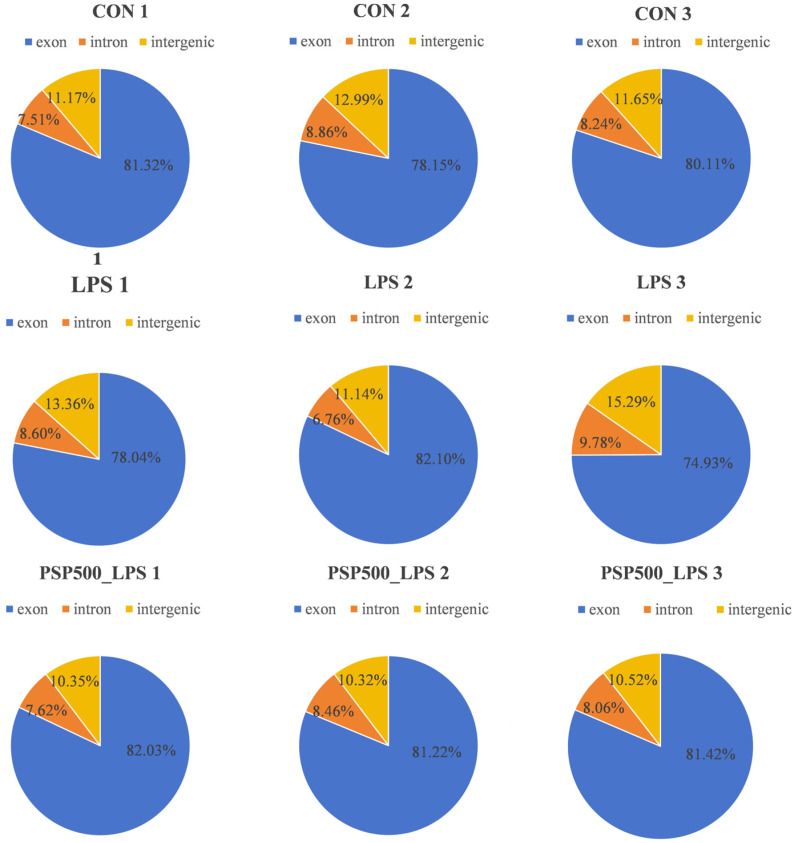
Distribution of sequenced reads across genomic regions for all samples.

We assessed the distribution of sequencing reads across genomic regions for each sample, finding similar percentages among different regions, which indicates comparability at the genomic level. This establishes a robust foundation for further gene expression analysis, variant detection, and functional annotation (Figure 5).

**Figure 6:**
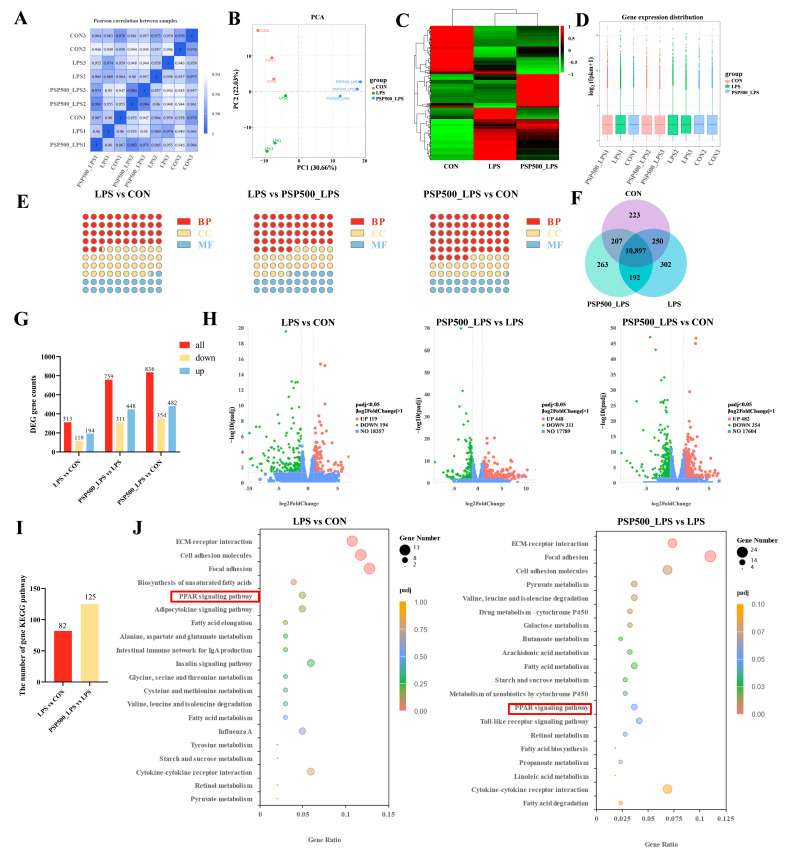
(**A**) Heatmap of inter-sample correlation. (**B**) Principal component analysis. (**C**) Differential gene clustering. (**D**) Box plots of gene expression distribution in samples. (**E**) GO enrichment analysis of the number of DEGs and the compositional ratio of GO entries of the genes in biological processes (BP), molecular functions (MF), and cellular components (CC) of the chick livers of each group, (**F**) Co-expression Wayne plots. (**G**) KEGG pathways of the DEGs in the livers of the chicks of each group and number of statistical plots. (**H**) Volcano plots of differentially expressed genes in the livers of chicks in each group. (**I**) Statistical plots of KEGG, and (**J**) bubble plots. The red boxes represents the shared pathway diagram between the two teams’ comparison combinations.

Expression of differential genes. The correlation coefficients among the CON, LPS, and PSP500_LPS groups were all above 0.92, confirming the reliability of our sequencing results (Figure 6A). Principal component analysis (PCA) showed that the nine samples were clearly divided into three parts in the PCA score plot of the chick liver transcriptome, indicating that there were significant differences in mRNA expression among the CON, LPS, and PSP500_LPS groups (Figure 6B). The transcript samples from the PSP-treated chicks clustered together, distinct from the LPS group and closer to the CON group, suggesting that PSP intervention significantly affects LPS-induced hepatic gene expression.

Additionally, a heatmap of differentially expressed genes was generated, displaying the sample names on the horizontal axis and the normalized Fragments Per Kilobase of transcript per Million mapped reads (**FPKM**) values on the vertical axis. FPKM is expressed as the expression level of the gene (Figure 6C). Red signifies higher expression levels, whereas green indicates lower expression levels. To assess mRNA expression levels and abundances, FPKM were calculated for each sample. Box plots were utilized to illustrate the distribution of gene expression levels across different samples (Figure 6D). The minimal gene dispersion and slight intra-group variations depicted in Figure 6D indicate a consistent FPKM distribution across samples, which is consistent with the results of the inter-group differential gene analysis.

A total of 10,897 differentially expressed genes were identified through a comparative analysis of the CON, LPS, and PSP500_LPS groups using the criteria of *p* < 0.05 and |log2(Fold Change)| > 1, as illustrated in the Venn diagram (Figure 6F).

GO functional clustering analysis of differentially expressed genes. Based on the screened DEGs, a total of 347 terms were enriched in the Gene Ontology (**GO**) database in the LPS group compared to the CON group, including 147 entries in the biological process (**BP**) group, 125 entries in the cellular component (**CC**) group, and 75 entries in the molecular function (**MF**) group. Compared with the PSP500_LPS group, the LPS group was enriched with 515 entries, with 231 entries in the BP group, 152 entries in the CC group, and 132 entries in the MF group. In comparison to the CON group, the PSP500_LPS group was enriched with 611 entries, including 336 entries in the BP group, 149 entries in the CC group, and 126 entries in the MF group (Figure 6E). 

KEGG pathway enrichment analysis of differentially expressed genes. We used RNA-Seq to identify DEGs in the liver tissues of chicks from the CON, LPS, and PSP500_LPS groups. In comparison to the CON group, the liver tissues of the LPS group exhibited 313 DEGs, among which 194 were up-regulated and 119 were down-regulated. In comparison to the LPS group, 759 DEGs were identified in the PSP500_LPS group, comprising 448 up-regulated and 311 down-regulated genes. When comparing the PSP500_LPS group to the CON group, we identified a total of 836 DEGs, including 482 up-regulated and 354 down-regulated genes (Figure 6G,H).

Through KEGG pathway enrichment analysis, we identified 82 significantly enriched signaling pathways in the LPS group compared to the CON group. Analysis of the PSP500_LPS group revealed 125 significantly enriched pathways compared to the LPS group (Figure 6I). The top 20 enriched pathways are presented in a bubble diagram (Figure 6J). Key pathways affected by LPS included the PPAR signaling pathway, focal adhesion, cell adhesion molecules, and ECM-receptor interaction. After PSP intervention, the primary impacted pathways included the PPAR signaling pathway, pyruvate metabolism, cell adhesion molecules, focal adhesion, and ECM-receptor interaction. These findings suggest that PSP may protect against liver damage by regulating lipid metabolism, modulating cell growth and proliferation, and inhibiting the expression of inflammatory factors.

We focused further on the PPAR signaling pathway, which was commonly involved in both comparisons, and identified 10 DEGs within it: *APOC3*, *ACAA1*, *HMGCS2*, *ACSBG2*, *ME1*, *ADIPOQ*, *FABP3*, *FABP4*, *SCD*, and *PCK1*.

**Figure 7:**
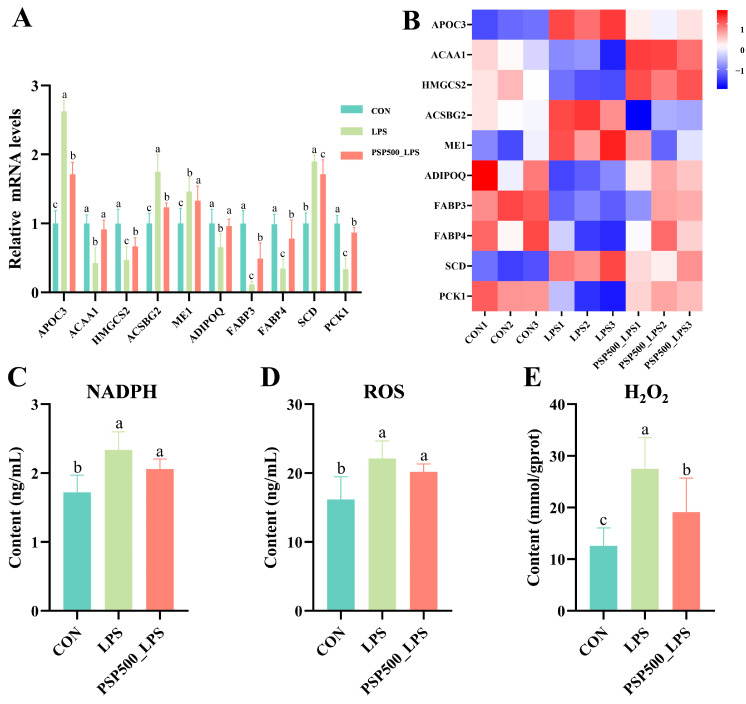
Differentially expressed genes in the PPAR signaling pathway. (**A**) Relative mRNA expression levels of *APOC3*, *ACAA1*, *HMGCS2*, *ME1*, *ADIPOQ*, *FABP3*, *FABP4*, *SCD*, and *PCK1* were determined by qPCR. (**B**) Heatmap of the nine significantly differentially expressed genes. (**C**–**E**) Chick liver NADPH, ROS, and H_2_O_2_ levels. Data columns labelled with different lowercase letters indicate statistically significant differences (*p* < 0.05).

Detection of differentially significant correlates in PPAR signaling pathway qPCR validation. The transcriptome analysis indicated that the relative mRNA expression of *ACAA1*, *HMGCS2*, *ADIPOQ*, *FABP3*, *FABP4*, and *PCK1* was significantly down-regulated in the LPS group compared to the CON group (*p* < 0.05). Conversely, the relative mRNA expression of *APOC3*, *ACSBG2*, *ME1*, and *SCD* was significantly up-regulated in the LPS group (*p* < 0.05). In the PSP500_LPS group, the relative mRNA expression levels of *ACAA1*, *HMGCS2*, *ADIPOQ*, *FABP3*, *FABP4*, and *PCK1* were notably higher compared to those in the LPS group (*p* < 0.05), while the expression of *APOC3*, *ACSBG2*, *ME1*, and *SCD* was significantly down-regulated (*p* < 0.05) (Figure 7A,B). The qRT-PCR analysis validated that the gene expression patterns were consistent with the transcriptome sequencing results, both in direction and magnitude, thereby validating the reliability of the transcriptome analysis.

LPS exposure significantly increased the levels of NADPH, ROS, and H_2_O_2_ in chick liver (*p* < 0.05). However, the addition of PSP significantly mitigated the LPS-induced increase in NADPH, ROS, and H_2_O_2_ levels (Figure 7C–E).

**Figure 8:**
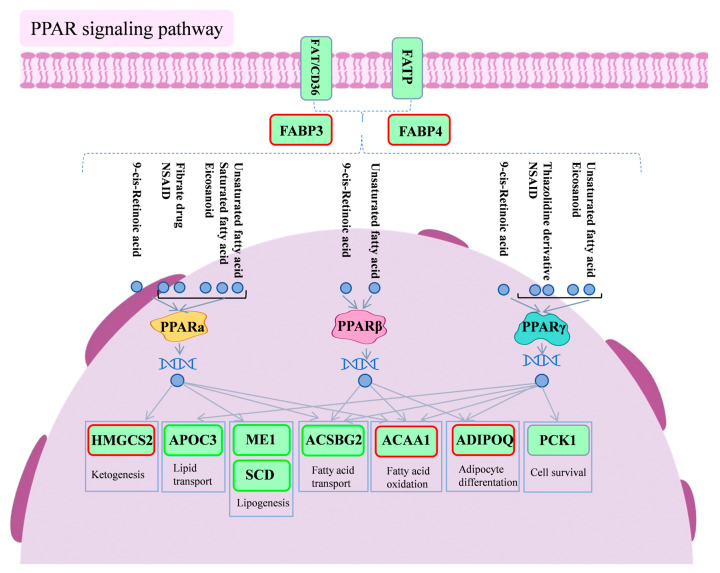
Diagram of PPAR signaling pathway. Red boxes indicate differential genes with increased expression in the PSP500_LPS group compared to the LPS group, and green boxes indicate differential genes with decreased expression in the PSP500_LPS group compared to the LPS group.

Figure 8 is a schematic diagram of the PPAR signaling pathway.

## 4. Discussion

The liver plays a crucial role in intrinsic immunity, and its histomorphological features serve as key indicators of health status [16]. LPS stimulation induces pro-inflammatory cytokine release from hepatic macrophages, causing liver injury characterized by oxidative stress, lobular structure disruption, inflammatory infiltration, and hepatocyte necrosis [17,18,19]. In this study, LPS-treated groups exhibited hepatic inflammatory infiltration. HE-stained sections revealed blurred hepatic cord structures, central vein congestion, and extensive hepatocyte necrosis. Liver and kidney parameters were significantly elevated in the LPS group, confirming successful liver injury model establishment via intraperitoneal LPS injection. Notably, supplementation with varying doses of PSP in drinking water alleviated liver injury to different extents. ALT and AST, key biomarkers of hepatic dysfunction, are released into the bloodstream due to increased hepatocyte permeability during liver injury, leading to elevated serum levels [20,21]. Our results demonstrate that PSP effectively reduces LPS-induced liver damage in chicks and maintains normal ALT and AST levels, indicating its protective role against LPS-induced liver injury in this model.

Antioxidant capacity is vital for maintaining animal health, as oxidative stress can trigger inflammation [22]. Under normal conditions, oxidative and antioxidant levels remain balanced. However, an imbalance can lead to excessive free radical production, such as ROS, causing oxidative stress and cellular damage [23]. LPS can induce the overproduction of free radicals, which attack cellular and organelle membranes, thereby reducing the activity of antioxidant enzymes like SOD, primarily localized in organelle membranes, and causing structural damage to these membranes [24]. Additionally, an increase in MDA levels serves as an indicator of heightened lipid peroxidation. In this study, LPS decreased SOD levels while increasing ROS and MDA content. In contrast, PSP reversed these effects, consistent with findings by Shen et al. [25]. These results suggest that PSP enhances antioxidant capacity in chicken liver and alleviates LPS-induced oxidative damage, offering new insights into antioxidant intervention strategies.

Peroxisome proliferator-activated receptors (PPARs) are a family of nuclear receptors that play a pivotal role in the regulation of lipid metabolism, inflammatory responses, and energy homeostasis. The PPAR family includes three isoforms: PPARα, PPARβ/δ, and PPARγ [26]. PPARα is highly expressed in the liver, heart, and kidneys. In vivo studies have demonstrated that the inflammatory response is prolonged in PPARα-deficient mice with exacerbated LPS-induced inflammation in the aorta, indicating PPARα’s role as a modulator of inflammation [27]. PPARβ/δ, widely expressed across tissues, regulates fatty acid oxidation and blood glucose levels. PPARγ is present in the liver, muscle, and immune cells [28] and possesses the capacity to inhibit inflammation, suppress tumor factors, and regulate immune responses, among other biological functions [29]. PPARγ agonists reduce VCAM-1 expression, inhibit monocyte and neutrophil adhesion, and mitigate chronic inflammation [30,31,32]. Activated PPARγ suppresses pro-inflammatory cytokine expression by inhibiting NF-κB and JAK-STAT signaling pathways, thereby reducing pro-inflammatory cytokines and enhancing anti-inflammatory cytokines [33,34,35]. These results are consistent with the findings of our study, which revealed an increase in pro-inflammatory cytokines IL-1β, IL-6, and TNF-α, along with a decrease in the anti-inflammatory cytokine IL-10 in the livers of chicks exposed to LPS. Notably, the introduction of PSP reversed these trends, suggesting that PSP intervention exerts an anti-inflammatory effect on liver tissue and influences the inflammatory response in the chick liver.

Studies have shown that *APOC3*, a key protein in lipid metabolism, is closely associated with inflammatory diseases. *APOC3* activates the NLRP3 inflammasome via the TLR2/NF-κB pathway, involving caspase-8 and TLR2/TLR4 dimerization, leading to inflammation and organ damage [36,37]. NLRP3 inflammasome activation is a critical step in the inflammatory cascade, causing tissue damage. In our study, LPS exposure significantly up-regulated *APOC3* expression, likely promoting NLRP3 inflammasome activation in chick livers and exacerbating hepatic inflammation and injury. Notably, the incorporation of PSP into the drinking water reversed the expression pattern of the *APOC3* gene and reduced inflammatory damage, further confirming the significant role of *APOC3* in the inflammatory response.

*HMGCS2* serves as a pivotal enzyme in the ketone body synthesis pathway and holds a crucial role in immunomodulation [38]. Reduced *HMGCS2* expression in ulcerative colitis patients may exacerbate intestinal inflammation. Research suggests *HMGCS2* can mitigate inflammation in intestinal epithelial cells by regulating PPARγ [39]. In our study, after intraperitoneal injection of LPS into chicks, we noted a decrease in the expression level of *HMGCS2* in the liver. This reduction in *HMGCS2* expression may diminish its regulatory effect on PPARγ, subsequently impairing the anti-inflammatory actions of PPARγ and leading to the persistence and exacerbation of inflammatory responses in liver tissues.

*ME1* is involved in various biochemical reactions within cells, particularly in the production of nicotinamide adenine dinucleotide phosphate (NADPH). NADPH serves as a key reductant for eliminating lipid peroxides and is essential for maintaining cellular redox homeostasis [40]. Reduced *ME1* activity decreases NADPH levels, weakening antioxidant defenses and leading to reactive oxygen species (ROS) accumulation, such as H2O2, and lipid peroxide buildup [41]. In this study, we observed that LPS significantly up-regulated *ME1* gene expression, suggesting that cells may attempt to boost NADPH production to enhance their antioxidant capacity. However, PSP supplementation in chick drinking water reduced *ME1* expression, decreased ROS production, inhibited lipid peroxide accumulation, and alleviated oxidative stress. We speculate that PSP may influence the downstream signaling or enzyme activity of *ME1* through specific pathways, enabling cells to maintain the effective utilization of NADPH despite reduced *ME1* expression, thereby reducing ROS production in liver tissue. This is supported by our measurements of NADPH and ROS levels in this study.

Fatty acid binding proteins (FABPs) are intracellular transporters of fatty acids, with *FABP3* and *FABP4* playing essential roles in glycolipid metabolism and inflammation regulation [42,43]. Inhibiting *FABP4* has been shown to improve lipid metabolism disorders and reduce chronic metabolic inflammation [44]. *FABP4* is expressed in macrophages, which play a crucial role in inflammation by being responsible for phagocytosing pathogens, damaged cells, and lipids and the release of inflammatory mediators [45]. The sequencing data revealed a notable decrease in the expression levels of *FABP3* and *FABP4* in the livers of chicks subjected to LPS induction. Notably, the addition of PSP reversed the decreasing trend of *FABP3* and *FABP4*, suggesting that PSP might be involved in up-regulating the expression of these proteins. As illustrated in the pathway diagram, FABPs are co-regulated by FAT/CD36 and FATP and act upstream of the PPAR signaling pathway.

Stearoyl-CoA desaturase (*SCD*) is a key enzyme in fatty acid desaturation, playing a vital role in fatty acid synthesis and metabolism [46]. Excessive saturated fatty acid intake can increase oxidative stress and inflammation, contributing to atherosclerosis [47,48]. In contrast, monounsaturated fatty acids exhibit anti-inflammatory effects and can help reduce inflammatory responses and oxidative stress damage [49]. Research has illuminated the pivotal role of PPARα activation in mitigating inflammation and enhancing vascular function. Its anti-inflammatory prowess is manifested through the prevention of lipid accumulation, the down-regulation of adhesion molecule expression, the inhibition of inflammatory cytokine production, and the obstruction of leukocyte adhesion and aggregation [50]. Experimental findings reveal that exposure to LPS leads to an up-regulation of *SCD* expression, which may promote fatty acid desaturation while simultaneously intensifying the activation of inflammatory cells and the secretion of inflammatory cytokines, thus exacerbating the inflammatory response. Conversely, the introduction of PSP markedly diminishes *SCD* expression, indicating its potential to inhibit *SCD* and manifest anti-inflammatory effects. The interplay among *SCD*, PPARα activation, LPS, and the influence of PSP weaves a complex regulatory tapestry that governs inflammatory responses and vascular functionality. Future investigations will further explore these intricate mechanisms, aiming to furnish scientific evidence for the treatment of related ailments.

## 5. Conclusions

The current research indicates that PSP may attenuate inflammation in LPS-induced liver injury by regulating the expression of differential genes, thereby reducing the levels of inflammatory factors and alleviating symptoms such as ballooning degeneration and cell swelling in the liver. These beneficial effects may be achieved through the activation of the PPAR signaling pathway, which is a key factor in suppressing hepatic inflammatory responses and oxidative stress. In summary, PSP ameliorated LPS-induced liver injury and reduced the inflammatory response and oxidative stress through a mechanism potentially related to the activation of the PPAR signaling pathway.

## Figures and Tables

**Table 1:** Nutrient levels of experimental animal base diet formula (air-dry basis, %).

Ingredient	Contents	Nutrient Levels	Contents
Corn	40.5	Metabolizable energy, MJ/kg	12.57
Soybean meal	21.65	Crude protein	20.5
Flour	16	Crude ash	7.5
Peanut meal	4	Crude fiber	2.75
CaHPO4	1.11	Ether extract	3.75
Corn protein	6.5	Calcium	1.13
Limestone	1.15	Total phosphorus	0.65
L-Lys	0.79	Methionine	0.56
NaCl	0.23	Lysine	1.25
Montmorillonite	0.3		
DL-Met	0.12		
Trace mineral premix ^1^	0.08		
Choline-Cl	0.08		
L-Thr	0.1		
Soybean oil	6.99		
BV25	0.4		
Total	100.00		

^1^ Premixes provide per kilogram of ration: VA 6141.5 IU; VD3 1681.4 IU; VE 30 IU; VK 1.91 mg; VB1 9.69 mg; VB2 4.01 mg; VB6 1.99 mg; VB12 0.01 mg; niacin 18.06 mg; calcium pantothenate 6.79 mg; folic acid 0.60 mg; biotin 0.07 mg; Fe 60.91 mg; Zn 66.75 mg; Cu 6.01 mg; Mn 68.3 mg; I 10.9 mg; Se 0.2 mg.

**Table 2:** Primer information.

Primer Name	Forward Sequence (5′→3′)	Reverse Sequence (5′→3′)	Accession No.
*APOC3*	TGCCTGCAGAAGAAGCCTCG	GACGGGCTCAAAAACCTCCT	NM_001302127.2
*ACAA1*	TGTGTATGTGCAGCAACCCGTAGT	GGCATTGCAATTTGGACAGAAGT	XM_046911759.1
*HMGCS2*	ATGTAGCTGACGGTGGACCT	TTCACGCCATCAGGAAGGTT	XM_422225.8
*ACSBG2*	GTGCCCACGCTGTGCTTAC	AGGAAACCTCTCCCTGGATGTC	XM_046933775.1
*ME1*	GGCTTGCTAGGGGAAATGA	AGCTGACTCTGACTAGGAAACTGT	NM_204303.2
*ADIPOQ*	GGAGCTGAGGGTGAAGTTTGA	GACACAGACTGGCAGCCAAA	NM_206991.2
*FABP3*	GCAGCACCTACCTCAACCAG	CTTACTGCGCGTCTTCTGGG	NM_001030889.2
*FABP4*	CTGGGTCTGGTTGGTGTGTTTG	GACAGCCATCCGCATCTTCTTC	NM_204290.2
*SCD*	TCTTCCTCCTCCCGCTTCTTCAC	AGAGATGGTGGTGTAGGCAGTGG	NM_204890.2
*PCK1*	AGCGAGATGTTGGCGATGAT	AAGTTGCCACACAGACCACA	NM_205471.2
*β-actin*	ATTGTCCACCGCAAATGCTTC	AAATAAAGCCATGCCAATCTCGTC	NM_205518.2

**Table 3:** Transcriptome sequencing and mapping quality analysis.

Sample	Raw Data (Read)	Valid Data (Read)	Valid Ratio (Reads)	Q30%	GC Content%
CON 1	48,873,026	47,319,450	96.82	93.5	48.36
CON 2	47,312,972	46,186,700	97.62	93.78	49.25
CON 3	47,216,436	46,031,068	97.49	93.01	48.32
LPS 1	47,241,592	45,907,894	97.18	93.88	47.39
LPS 2	49,986,422	48,787,892	97.60	94.07	48.51
LPS 3	48,556,376	47,157,778	97.12	93.57	47.39
PSP500_LPS 1	47,996,216	46,254,826	96.37	94.02	47.19
PSP500_LPS 2	47,648,792	45,984,124	96.51	94.07	46.76
PSP500_LPS 3	46,155,614	44,916,940	97.32	94.33	46.8

## Data Availability

The data analyzed during the current study are available from the corresponding author on reasonable request.

## Abbreviations

The following abbreviations are used in this manuscript:

PSP	*Polygonatum sibiricum* polysaccharide
LPS	Lipopolysaccharide
PPAR	Peroxisome proliferator-activated receptor
ROS	Reactive oxygen species
MDA	Malondialdehyde
SOD	Superoxide dismutase
ALT	Alanine aminotransferase
AST	Aspartate aminotransferase
TP	Total protein
CAT	Catalase
GSH-Px	Glutathione peroxidase
GO	Gene Ontology
BP	Biological process
CC	Cellular component
MF	Molecular function
